# Editorial: Leadership, alliance, and the 4th industrial revolution

**DOI:** 10.3389/fpsyg.2023.1258431

**Published:** 2023-08-04

**Authors:** Jeoung Yul Lee, Seong-jin Choi, Alfredo Jiménez

**Affiliations:** ^1^School of Business Management, Hongik University, Sejong, South Korea; ^2^School of Business, Hanyang University, Seoul, South Korea; ^3^Department of Management, Kedge Business School, Talence, France

**Keywords:** interpersonal relationship, interorganizational relationship, leadership, industry 4.0, alliance, artificial intelligence

The Fourth Industrial Revolution (4IR) is characterized by the integration of sophisticated technologies such as the Internet of Things (IoT), artificial intelligence (AI), and big data into all aspects of society and business. This transformation is profoundly influencing corporate management, behavior, and performance, as the papers in this Research Topic have shown. One of the key ways that 4IR is affecting enterprise relationships is through the increased use of AI. AI is being used to automate many tasks and processes, leading to improved efficiency and productivity. AI is also being used to analyze data and make predictions, which can help businesses make better decisions and improve their overall performance. 4IR technologies are also affecting the way that organizations interact and collaborate with each other through the development of new enterprise networks. These networks are characterized by integrating sophisticated technologies, e.g., IoT, AI, big data, etc., and can lead to improved efficiency, productivity, and performance. These networks can also be utilized to share data and resources that help businesses make better decisions and improve their overall performance. For example, Liu and Huang explore the role of participatory leadership in the construction of virtual teams. They argue that participatory leadership, characterized by providing greater space for the development of team members, is conducive to the expansion of the scale of virtual teams. This is particularly relevant in a decentralized and non-authoritative environment, such as that created by 4IR technologies. However, they also note that losing management is not conducive to building a reasonable structure of team members under participatory leadership. The balance between cooperation and competition, organizational trust, and the leadership incentive way are all crucial factors that influence the construction of virtual teams. In the context of 4IR, Liu and Huang research provides valuable insights into how leadership styles and organizational structures need to adapt to harness the potential of new technologies. Their work underscores the need for a shift in managerial focus from controlling and directing employees to enabling and empowering them to use these technologies effectively.

4IR technologies are fundamentally transforming our work practices, and remote work is a prominent example. Remote work has wide-ranging implications not only on job performance but also on organizations and structures. In this regard, the following papers analyze remote work, which is being facilitated by technological advancements, from various perspectives. From this view, Meiryani et al. scrutinizes the influence of remote work on the overall performance of organizations. It elucidates how remote work, enabled by 4IR technologies, has led to enhanced productivity and employee satisfaction, while also resulting in substantial cost savings. However, it also brings to light the complexities of managing remote teams, emphasizing the need for effective communication and fostering a sense of community. Simon et al. delves into the correlation between remote work and employee engagement. The research suggests that remote work can foster higher levels of employee engagement, provided that organizations offer the necessary support and resources. This includes the provision of appropriate technology and the cultivation of a culture that embraces flexible working arrangements. Prasad et al. investigates the impact of remote work on occupational stress. The findings reveal that elements of remote working, such as employee self-proficiency and teamwork, have a significant bearing on job satisfaction, intrinsic and extrinsic motivation, and employee performance. However, the study also confirms that occupational stress, a potential downside of remote working, can adversely affect these factors. Collectively, these papers underscore the transformative potential of remote work, a trend that has been accelerated by the 4IR. They highlight the necessity for organizations to adapt their practices and cultures to this new paradigm, ensuring they can harness the benefits while mitigating the challenges. As we continue to navigate the 4IR, gaining a comprehensive understanding of the dynamics of remote work will be crucial for organizations aiming to thrive in the future of work.

The Fourth Industrial Revolution (4IR) is reshaping the landscape of management theory and practice, challenging traditional models and introducing new paradigms that reflect the rapid technological changes we are witnessing. The two papers in this issue, by Jiang et al. and Huang et al., offer fresh perspectives on this transformation, providing new theoretical models that reflect these changes. Jiang et al. present a novel research model that integrates several well-established theories, including network theory, dynamic capability theory, and upper echelons theory. Their model explores the relationship between alliance capability and technology innovation performance, a critical aspect of 4IR. They propose that the relationship between alliance capability and innovation outcome is mediated by the network, a fresh perspective that advances our understanding of alliance theory in the context of 4IR. This mediated-moderating model offers a new lens through which to view the role of alliances in driving innovation, a key element of the 4IR.On the other hand, Huang et al. delve into the role of leadership in the context of Research and Development (R&D). They propose a unique theoretical model that examines how leadership roles evolve to strengthen teams' innovation and creativity, a crucial factor in the 4IR. They argue that the generation of creative ideas influences the leadership process and can enhance an organization's competitive advantage. This perspective challenges traditional views of leadership, suggesting that in the context of 4IR, leaders must evolve to foster creativity and innovation. Both papers highlight the significant impact of 4IR on conventional management theory. The use of AI and automation, for example, can lead to flatter organizational structures and decentralized decision-making, challenging managers' ability to maintain control and oversight. Similarly, the use of big data and IoT necessitates a shift in managerial focus from controlling and directing employees to enabling and empowering them to use these technologies. In other words, the 4IR is not just a technological revolution; it is also a management revolution. As these papers demonstrate, to thrive in the 4IR, organizations must rethink their management models and practices, embracing new theories and perspectives that reflect the changing technological landscape.

In view of the findings of this Research Topic, 4IR is having a significant impact on how enterprises interact and collaborate.Technology has always been a critical determinant of multiple firm outcomes (including efficiency, performance, competition, and survival), with a key role on economic development, and therefore it has attracted plenty of scholarly attention. Yet, the recent developments offered by 4IR are very significant and profound that we do not have a comprehensive understanding of all its repercussions and ramifications. Therefore, there needs to be more diverse theoretical and empirical evolutions for future research based on interdisciplinary perspectives. We believe that this would be fruitful not only for academics, as the 4IR makes it necessary to refine existing theories or even coming up with new ones, but also for practitioners as well as policy-makers given the magnitude of the economic impact of these new technologies. The studies included in this Research Topic represent a first step into this direction, not only in terms of the topics studied, the theoretical advacements suggested, and the relevance of the findings, but also the suggestions for future research. We hope their insights inspire scholars to increasingly pursue this research direction, allowing a deeper understanding of the agenda.

## Author contributions

JL: Supervision, Writing—original draft, Writing—review and editing. S-jC: Writing—original draft, Writing—review and editing. AJ: Writing—review and editing.

